# Management of gastro-bronchial fistula complicating a subtotal esophagectomy: a case report

**DOI:** 10.1186/1471-2482-9-20

**Published:** 2009-12-24

**Authors:** James D Martin-Smith, John O Larkin, Finbar O'Connell, Narayanasamy Ravi, John Vincent Reynolds

**Affiliations:** 1 Department of Clinical Surgery, St James's Hospital and Trinity College Dublin, Dublin, Ireland; 2 Department of Respiratory Medicine, St James's Hospital and Trinity College Dublin, Dublin, Ireland

## Abstract

**Background:**

The development of a fistula between the tracheobronchial tree and the gastric conduit post esophagectomy is a rare and often fatal complication.

**Case presentation:**

A 68 year old man underwent radical esophagectomy for esophageal adenocarcinoma. On postoperative day 14 the nasogastric drainage bag dramatically filled with air, without deterioration in respiratory function or progressive sepsis. A fiberoptic bronchoscopy was performed which demonstrated a gastro-bronchial fistula in the posterior aspect of the left main bronchus. He was managed conservatively with antibiotics, enteral nutrition via jejunostomy, and non-invasive respiratory support. A follow- up bronchoscopy 60 days after the diagnostic bronchoscopy, confirmed spontaneous closure of the fistula

**Conclusions:**

This is the first such case where a conservative approach with no surgery or endoprosthesis resulted in a successful outcome, with fistula closure confirmed at subsequent bronchoscopy. Our experience would suggest that in very carefully selected cases where bronchopulmonary contamination from the fistula is minimal or absent, there is no associated inflammation of the tracheobronchial tree and the patient is stable from a respiratory point of view without evidence of sepsis, there may be a role for a trial of conservative management.

## Background

The development of a fistula between the tracheobronchial tree and the gastric conduit post esophagectomy is a rare and often fatal complication. Most fistulae occur from direct communication between a dehisced anastomosis and adjacent bronchus. Anastomotic leaks are responsible for approximately 40% of post-esophagectomy deaths [1]. Clinically apparent thoracic anastomotic leaks and fistulae are associated with a high rate of mortality despite advances in critical care and endoprostheses [2]. We present herein a particularly rare case, a fistula from the left main bronchus into a cervical esophagogastric anastomosis, and discuss the presentation and the approach to successful conservative management.

## Case Presentation

A 68 year old man presented with a five month history of progressive dysphagia and weight loss of 5 kg. An adenocarcinoma arising in Barrett's epithelium in the lower third of the esophagus was diagnosed, and staging including CT-PET and endoscopic ultrasound suggested clinical T3N1 M0 staging. He was treated with a standard regimen of neoadjuvant chemoradiotherapy prior to esophagectomy [3]. At surgery, extensive fibrosis was evident, and an en-bloc resection was performed including thoracic duct, part of pericardium, and mediastinal lymph node dissection including complete clearance of the sub-carinal nodes, and a cervical hand-sewn anastomosis was fashioned. Pathology revealed a ypT3N1 tumor, with clear margins, and 7 of 30 glands involved by tumor.

**Figure 1:**
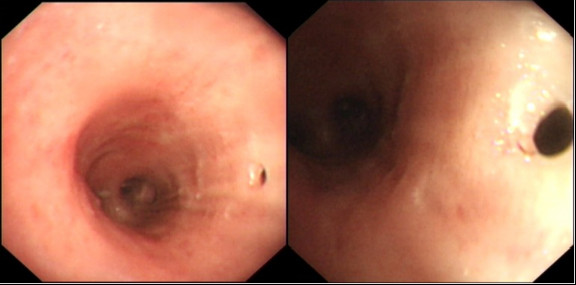
**Bronchoscopic appearance (day 16 post-op) of a fistulous opening in the posterior aspect of the left main bronchus, communicating with esophagogastric anastomosis**.

**Figure 2:**
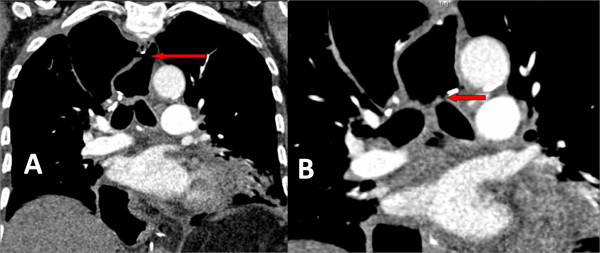
**CT thorax showing the mediastinal collection of air with a single, long fistulous communication between the esophagogastric anastomosis located the neck (A) and the left main bronchus (B)**.

On day four postoperatively he had a neutrophil leucocytosis of 15 × 10^9^/L and evidence of left basal consolidation. This persisted despite antibiotics, and a CT of thorax demonstrated no other abnormalities. He was managed on the ward, and his FiO_2 _varied from 0.4 to 0.6. Aspiration pneumonia was considered possible, and his nasogastric tube was left *in situ*. His neck wound was dry with no signs of inflammation or leakage. On day 14 the nasogastric drainage bag dramatically filled with air, and this persisted throughout the day and succeeding days, but without deterioration in respiratory function or evidence of progressive sepsis. A fiberoptic bronchoscopy was performed on day 16 which demonstrated bubbling at a gastro-bronchial fistula in the posterior aspect of the left main bronchus (Fig. 1). An endoscopy revealed a healthy gastric tube but a tiny area of granulation tissue in the anterior portion of the anastomosis, the assumed site of fistula communication. A CT scan of the thorax demonstrated air in the mediastinum and the gastric conduit (Fig. 2)

**Figure 3:**
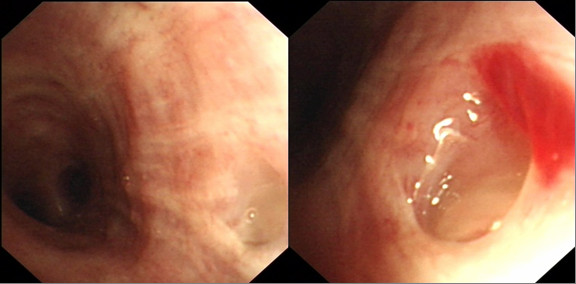
**Follow-up bronchoscopic evaluation 60 days after the prior examination shows closure of the fistulous opening**.

He was managed conservatively with antibiotics, enteral nutrition via a jejunostomy, and non-invasive respiratory support in the form of humidified oxygen via face mask and chest physiotherapy. The huge amounts of air in the nastogastric bag persisted for a further 9 days. When it subsided, he was introduced to oral diet and progressed well and was discharged. A follow- up bronchoscopy 60 days after the diagnostic bronchoscopy, confirmed spontaneous closure of the fistula (Fig. 3).

## Conclusions

The development of a fistula between the gastric tube and the tracheobronchial tree represents a very rare but potentially catastrophic complication after esophagogastrostomy for esophageal carcinoma. The commonest cause is a leak from the esophagogastric intrathoracic anastomosis with subsequent mediastinal abscess and rupture into the posterior wall of the tracheobronchial tree. Anastomotic leaks are responsible for approximately 40% of post-esophagectomy deaths [1]. Clinically apparent thoracic anastomotic leaks and fistulae are associated with a high rate of mortality despite advances in critical care and endoprostheses [2].

Most anastomotic leaks result from gastric ischemia, but this does not appear to have been the problem with our patient. Potential mechanisms in this particular case include the rendering vulnerable or ischemic of the tracheobronchial tissue by neoadjuvant chemoradiotherapy, combined with sharp dissection to radically remove all sub-carinal nodal tissue, with consequent injury and delayed rupture. This is the first case to our knowledge to present in this fashion, where the primary source appears to be the bronchus with the secondary consequence at the anastomotic site. Generally, gastro-bronchial fistulae may present in the early postoperative period or relatively late in the follow-up, with management strategy influenced by the site and size of the fistula, the underlying cause, and the clinical presentation [4,5]. It has previously been postulated that untreated gastro-bronchial fistula is usually fatal due to chronic pulmonary sepsis and that conservative treatment is inadvisable [6]. However, surgery is fraught with high morbidity in these patients.

The commonest mode of presentation is cough after swallowing, dyspnea, fever and recurrent pneumonia [7]. These are non-specific symptoms in the post-esophagectomy period. In this case, the dramatic presentation of a naso-gastric bag filling every two hours with air made the diagnosis clinically obvious, and bronchoscopy established the source. Stenting of the bronchus was considered, but the site of fistulation did not lend itself easily to an endo-bronchial prosthesis. Endoscopy of the anastomosis and stomach helped to establish that the stomach was healthy, and predict that this would heal as long as the bronchial leak settled. Self-expanding esophageal endo-prostheses have been used successfully in patients with anastomotic leaks after esophageal surgery [8], usually intra-thoracic leaks. Stent migration has been reported in as many as 37.5% of patients. In this case, the high anastomosis, 3 cm below the cricopharyngeus, and a normal diameter nonstenotic anastomosis were considered to predict a high risk of stent failure so stenting was not seriously entertained.

To our knowledge, this is the first report of successful conservative management of a gastro-bronchial fistula complicating a subtotal esophagectomy. Our experience would suggest that in very carefully selected cases where bronchopulmonary contamination from the fistula is minimal or absent, there is no associated inflammation of the tracheobronchial tree and the patient is stable from a respiratory point of view without evidence of sepsis, there may be a role for a trial of conservative management.

In conclusion, this is the first experience of this high-volume center with such a complication, and to our knowledge the first such case reported. The case highlights how a fistula that is unlikely to have been caused by contamination will resolve itself, and that a conservative approach with no surgery or endoprosthesis resulted in a successful outcome.

## Consent

Written informed consent was obtained from the patient for publication of this case report and any accompanying images. A copy of the written consent is available for review by the Editor-in-Chief of this journal.

## Competing interests

The authors declare that they have no competing interests.

## Authors' contributions

**JMS **was involved in the postoperative clinical management and drafted the manuscript; **JL **was involved in the postoperative clinical management and drafted the manuscript; **FOC **provided postoperative respiratory consultation and performed the bronchoscopies; **NR **performed the surgery and oversaw the writing of the manuscript; **JVR **performed the surgery, oversaw patient management and edited the manuscript. All authors read and approved the final manuscript.

## Pre-publication history

The pre-publication history for this paper can be accessed here:

http://www.biomedcentral.com/1471-2482/9/20/prepub
